# Identification and Characterization of Glutathione S-transferase Genes in *Spodoptera frugiperda* (Lepidoptera: Noctuidae) under Insecticides Stress

**DOI:** 10.3390/toxics11060542

**Published:** 2023-06-19

**Authors:** Ahmed A. A. Aioub, Ahmed S. Hashem, Ahmed H. El-Sappah, Amged El-Harairy, Amira A. A. Abdel-Hady, Laila A. Al-Shuraym, Samy Sayed, Qiulan Huang, Sarah I. Z. Abdel-Wahab

**Affiliations:** 1 Plant Protection Department, Faculty of Agriculture, Zagazig University, Zagazig 44511, Egypt; 2 Stored Product Pests Research Department, Plant Protection Research Institute, Agricultural Research Center, Sakha, Kafr El-Sheikh 33717, Egypt; ashashem2014@gmail.com; 3 Department of Genetics, Faculty of Agriculture, Zagazig University, Zagazig 44511, Egypt; ahmed_elsappah2006@yahoo.com; 4 School of Agriculture, Forestry and Food Engineering, Yibin University, Yibin 644000, China; Aglaia_1988@163.com; 5 Unit of Entomology, Plant Protection Department, Desert Research Center, Mathaf El-Matariya St. 1, El-Matariya, Cairo 11753, Egypt; 6 Department of Integrated Pest Management, Plant Protection Institute, Hungarian University of Agriculture and Life Sciences, Páter Károly utca 1, 2103 Gödöllő, Hungary; 7 Economic Entomology Department, Faculty of Agriculture, Mansoura University, Mansoura 35516, Egypt; 8 Department of Biology, College of Science, Princess Nourah Bint Abdulrahman University, P.O. Box 84428, Riyadh 11671, Saudi Arabia; 9 Department of Economic Entomology and Pesticides, Faculty of Agriculture, Cairo University, Giza 12613, Egypt; 10 Department of Science and Technology, University College-Ranyah, Taif University, P.O. Box 11099, Taif 21944, Saudi Arabia

**Keywords:** glutathione S-transferase, gene expression, phylogenic tree, molecular docking, insecticides

## Abstract

Insect glutathione S-transferases (GSTs) serve critical roles in insecticides and other forms of xenobiotic chemical detoxification. The fall armyworm, *Spodoptera frugiperda* (J. E. Smith), is a major agricultural pest in several countries, especially Egypt. This is the first study to identify and characterize GST genes in *S. frugiperda* under insecticidal stress. The present work evaluated the toxicity of emamectin benzoate (EBZ) and chlorantraniliprole (CHP) against the third-instar larvae of *S. frugiperda* using the leaf disk method. The LC_50_ values of EBZ and CHP were 0.029 and 1.250 mg/L after 24 h of exposure. Moreover, we identified 31 GST genes, including 28 cytosolic and 3 microsomal SfGSTs from a transcriptome analysis and the genome data of *S. frugiperda*. Depending on the phylogenetic analysis, sfGSTs were divided into six classes (delta, epsilon, omega, sigma, theta, and microsomal). Furthermore, we investigated the mRNA levels of 28 GST genes using qRT-PCR under EBZ and CHP stress in the third-instar larvae of *S. frugiperda*. Interestingly, SfGSTe10 and SfGSTe13 stood out with the highest expression after the EBZ and CHP treatments. Finally, a molecular docking model was constructed between EBZ and CHP using the most upregulated genes (SfGSTe10 and SfGSTe13) and the least upregulated genes (SfGSTs1 and SfGSTe2) of *S. frugiperda* larvae. The molecular docking study showed EBZ and CHP have a high binding affinity with SfGSTe10, with docking energy values of −24.41 and −26.72 kcal/mol, respectively, and sfGSTe13, with docking energy values of −26.85 and −26.78 kcal/mol, respectively. Our findings are important for understanding the role of GSTs in *S. frugiperda* regarding detoxification processes for EBZ and CHP.

## 1. Introduction

The activity of detoxifying enzymes is necessary for an insect to survive toxic surroundings such as insecticides [1]. The process of cellular detoxification in insects can be separated into three phases: phase I, phase II (involving metabolizing enzymes), and phase III (involving transporters) [2]. Cytochrome P450 monooxygenase, glutathione S-transferase (GST), and carboxylesterase (CarE) are the primary enzymes involved in phase I and phase II detoxification processes [3], whereas phase III is dominated by ATP-binding cassette (ABC) transporters [4,5,6,7].

In insects, one of the most important detoxification enzymes in phase II is the GST family of multifunctional enzymes. GSTs are known to catalyze the nucleophilic attack of the sulfhydryl group of reduced glutathione (GSH) on electrophilic centers of xenobiotic compounds, including insecticides [8,9]. GSTs are categorized into four major protein types based on cellular locations, including cytosol, microsomes, mitochondria, and bacterial Fosfomycin-resistant proteins [8,10,11,12]. Only the first two groups have been found in insects thus far [13]. More genes are present in cytosolic GSTs than in microsomal GSTs [14]. GSTs in cytosols can either function as homodimers or heterodimers and typically include 200–250 amino acids. The six primary classes of insect cytosolic GSTs are delta, epsilon, omega, sigma, theta, zeta, and unclassified genes [15]. Microsomal GSTs are membrane-bound proteins that function as trimmers, and they typically have 150 amino acid residues [16]. Microsomal GSTs are membrane-associated proteins in eicosanoid and glutathione metabolism (MAPEG family), which are crucial for reducing lipid peroxidation and xenobiotic detoxification [17]. Reports correlating high levels of GST with high resistance to insecticides do exist for many insects [13,18]. GSTs confer insecticide resistance directly through metabolism or sequestration or indirectly by protecting against oxidative stress induced by synthetic insecticides [19]. For example, upregulated GSTu1 in several CHP-resistant *Plutella xylostella* strains contributed to CHP resistance [20]. Several GSTs have been implicated in resistance to organophosphates (OPs) in *Musca domestica* [21]. A biochemical study of *Anopheles gambiae* indicated that DDT resistance is associated with both quantitative and qualitative changes in multiple GST enzymes [22].

*Spodoptera frugiperda* (J. E. Smith) (Lepidoptera: Noctuidae), a natural species of tropical and subtropical origin in the Western Hemisphere, is a damaging pest in maize. Its wide host range includes corn, wheat, cotton, soybean, cabbage, and potatoes [23,24,25]. The early 21st century saw the introduction of this insect, which is indigenous to the Western Hemisphere, Africa, Asia, and Oceania [26]. The high reproduction rate, extensive migration, great dispersal ability, and vigorous flight (up to 500 km before oviposition) of *S. frugiperda* are factors contributing to its economic significance [27]. Since the fall armyworm was introduced to the Eastern Hemisphere and quickly spread from western Africa to southeastern Asia, these traits have become a global concern [23]. The first significant *S. frugiperda* infestations in Africa were discovered in southwestern Nigeria in 2016 [28]. In 2018, the Food and Agriculture Organization in Egypt declared it a global pest that requires quarantine. The first occurrence was in maize fields in the Upper Egypt Governorates in 2019 [29]. *S. frugiperda* can swiftly damage a maize crop because of its intense feeding, and if it is not promptly controlled, it may also destroy other crops. Following an *S. frugiperda* invasion, Brazil’s corn yield decreased by 34%, and the annual loss brought on by *S. frugiperda* is USD 400 million [30]. *S. frugiperda* control relies intensively on chemical insecticides, prompting resistance to many classes of insecticides [31,32]. Currently, *S. frugiperda* is among the top 15 most resistant insect pest species worldwide [33]. The resistance mechanism of insects toward insecticides comprises two main aspects, including detoxification enzyme activity upregulation and target-induced decreased sensitivity [34]. In *S. frugiperda*, enhanced glutathione transferase activity is associated with the degradation of Fluxametamide [35]. Another study showed that the overexpression of GSTs was involved in *S. frugiperda* developing resistance to pyrethroids, organophosphorus, and carbamate pesticides [34]. Consequently, *S. frugiperda* is considered one of the most dangerous insects and can cause heavy losses in the economic crops of Egypt.

Given the seriousness of *S. frugiperda* and the speed of its spread, it is necessary to intervene quickly with chemical pesticides. CHP and EBZ, which target ryanodine receptors and glutamate-gated chloride channel receptors, respectively, were found to be effective against *S. frugiperda*. In 2022–2023, the Egyptian Ministry of Agriculture suggested that CHP and EBZ be used to control *S. frugiperda*. Here, we hypothesize that GSTs play a role in *S. frugiperda* larva detoxification, suggesting a potential focus for further investigation into integrated pest management techniques. Thus, we aimed to evaluate the LC_50_ values of CHP and EBZ against 3rd instar *S. frugiperda* larvae. Moreover, we identified and characterized 31 GST genes (SfGSTs) in *S. frugiperda* using previously released transcriptome datasets [36] and genome data (InsectBase http://www.insect-genome.com (accessed on 30 April 2023). Furthermore, we determined the expressions of 28 out of 31 GST genes in S. frugiperda under insecticide stress using qRT-PCR. Finally, molecular docking was performed to clarify putative interactions between the proteins and tested insecticides.

## 2. Materials and Methods

### 2.1. S. frugiperda Rearing

*S. frugiperda* were collected from infested maize fields in the Assuit Governorate, Upper Egypt (27.2134° N, 31.4456° E) and then reared at the Plant Protection Research Institute, Agricultural Research Centre, Giza, Egypt, at 25 ± 3 °C and 70 ± 10% relative humidity and a photoperiod of 16:8 (L:D) h, without being exposed to insecticides. The larvae were put in 20 mL plastic cups and fed fresh corn leaves. The grown-up pupae were collected and kept in a plastic container inside a 30 cm × 30 cm × 30 cm rearing cage. Water was added to fresh plant leaves before they were placed in an egg-laying chamber. The larvae were moved for study once they reached the third larval age.

### 2.2. Bioassay of Tested Insecticides against Third-Instar S. frugiperda Larvae

Technical-grade EBZ (99.4%; catalog number: P-996S) and CHP (95%; catalog number: P-952S) were obtained from Sigma-Aldrich, China. The effectiveness of EBZ and CHP against *S. frugiperda* third larval instars in varied doses was assessed using the leaf disk method [37]. Briefly, active-ingredient insecticides were first dissolved in acetone. Fresh maize leaves were chopped into leaf discs that measured 0.5 cm × 0.5 cm and submerged in different concentrations of EBZ (0.005, 0.01, 0.02, 0.04, 0.08 mg/L) and CHP (0.25, 0.50, 1, 2, and 4 mg/L) for 10 s. Leaves coated with an equal amount of acetone were provided as a control. Then, the leaf strips were placed in a tray and allowed to air dry. A 24-well plate was filled with dried corn leaves. The third-instar larvae were transplanted to a 24-well plate after fasting for 12 h. A total of 30 larvae were used for each concentration. Each treatment had 3 replicates under carefully controlled settings (25 ± 3 °C) for 24 h. After 24 h, mortality was recorded. Dead larvae were those that did not move when touched by a brush. Mortality was calculated, and the LC_50_ value of each insecticide was calculated, as well as a 95% confidence interval.

### 2.3. Quantitative Real-Time (PCR qRT-PCR) Analysis

Following a 24-h feeding period on corn leaves with control and insecticide-treated larvae, the LC_50_ values were recorded for the live larvae and control, and they were collected for RNA extraction. Fifteen treated and control *S. frugiperda* larvae with three replicates were used to measure gene expression. Total RNA was isolated using RNAiso Plus reagent (Takara, Dalian, China) and treated with RNase-free DNase I (Takara, Dalian, China) to remove potential contaminants from genomic DNA. The quality and concentration of RNA were determined via agarose gel electrophoresis and a NanoDrop 2000 spectrophotometer (Thermo Scientific, Wilmington, CA, USA). First-strand cDNA was reverse-transcribed using the TransScript First-Strand cDNA Synthesis SuperMix (Transgen, Beijing, China). qRT-PCR primers were designed with Primer 3 Plus for 28 GST genes per previously published transcriptome datasets [36], and Table S1 contains a list of all the primer sequences utilized in this investigation. The internal reference genes chosen for this study were elongation factor 1-alpha (EF1) and ribosomal protein S18 (RPS18) [38], and the qRT-PCR was performed using an SYBR Green qPCR Master Mix kit (Catalog number: 4309155, Takara, Japan) with a 240 Light Cycler 480 II system (Roche Diagnostics, Mannheim, Germany) under the following parameters: 95 °C for 30 s, 40 cycles at 95 °C for 5 s, and 60 °C for 20 s. The 2^−ΔΔCt^ technique was used to calculate the relative quantification of gene expression (Livak and Schmittgen 2001).

### 2.4. Bioinformatics Analyses

cDNA sequences for GSTs were obtained from *S. frugiperda* transcriptome databases that were previously made available [36], as well as from a genome (InsectBase http://www.insect-genome.com (accessed on 30 April 2023). GST sequences from typical insect species were downloaded, including *Drosophila melanogaster*, *Spodoptera litura*, *Pieris rapae*, *Nilaparvata lugens*, *Sogatella furcifera*, *Leptinotarsa decemlineata*, *Bombyx mori*, and *Tribolium castaneum* from the National Centre for Biotechnology Information (NCBI) (https://www.ncbi.nlm.nih.gov/ (accessed on 30 April 2023) as queries using the TBLASTN algorithm in the Basic Local Alignment Search Tool (BLAST) program (http://blast.ncbi.nlm.nih.gov/blast.cgi (accessed on 30 April 2023) with a cut-off E-value of 1 × 10^−5^ [39]. After manually deleting duplicated sequences, we combined the GST genes found in the two datasets. The neighbor-joining approach with the pair-wise deletion option was used with the MEGA6.0 software to generate a phylogenetic tree [40].

### 2.5. In Silico Molecular Docking Assay

All modeled protein structures (SfGSTe10, SfGSTe13, SfGSTs1, and SfGSTe2) were downloaded from the National Center for Biotechnology Information (NCBI) server to construct 3D models. To create a more acceptable structural template for trustworthy theoretical 3D models, Swiss-model tools supplied the sequences of all proteins. Ramachandran’s plot (PROCHECK analysis) was then used to analyze and validate these models. Structure models of all proteins and their active sites (pockets) were downloaded in PDB format and imported into the Molecular Operating Environment (MOE) 2014.13 software (Chemical Computing Group Inc., Montreal, QC, Canada) [41]. The heteroatoms and crystallographic water molecules were eliminated from the protein after the protein’s missing hydrogen chemistry was restored [42].

Ligand selection, CHP, and EBZ were created using the Chem Draw Professional 15 Builder module. Before starting the docking process, the ligands were reduced using the CHARM m 99 force field. Three-dimensional (3D) structures were then constructed, duplicates were removed, and bonds were added. The ligands were made flexible and manually placed inside the catalytic site cavity of the enzyme model after all the default parameters were established and the minimal energy structures were obtained. A full-force field was used to investigate the binding energy, and scoring functions that generated free-binding interaction energies based on molecular force field terms were used to assess the ligand and protein’s affinity. At the conclusion of the docking, the best ligand interaction was investigated and evaluated using scoring functions and root-mean-square deviation (RMSD) computations [43].

### 2.6. Statistical Analysis

Probit analyses were used to determine LC_50_ values and 95% confidence intervals with the aid of the SPSS software (version 19.0, SPSS Inc., Chicago, IL, USA, 2003). The expression patterns based on qRT-PCR were examined using the 2^−∆∆Ct^ technique. The significance of differences between patterns was assessed using a one-way ANOVA with PASW Statistics and Duncan’s multiple range test. The outcomes were displayed using the relative mRNA expressions’ mean and standard deviations.

## 3. Results

### 3.1. Insecticidal Activity of Tested Insecticides against the Third Larval Instars of S. frugiperda

Table 1 shows the bioassay results of the leaf disk method for the two studied insecticides (CHP and EBZ) against third-instar *S. frugiperda* after one day of treatment. The LC_50_ values were 0.029 mg/L for EBZ and 1.250 mg/L for CHP after 24 h of treatment for the third-instar *S. frugiperda*.

### 3.2. Identification and Classification of S. frugiperda GSTs

**Figure 1:**
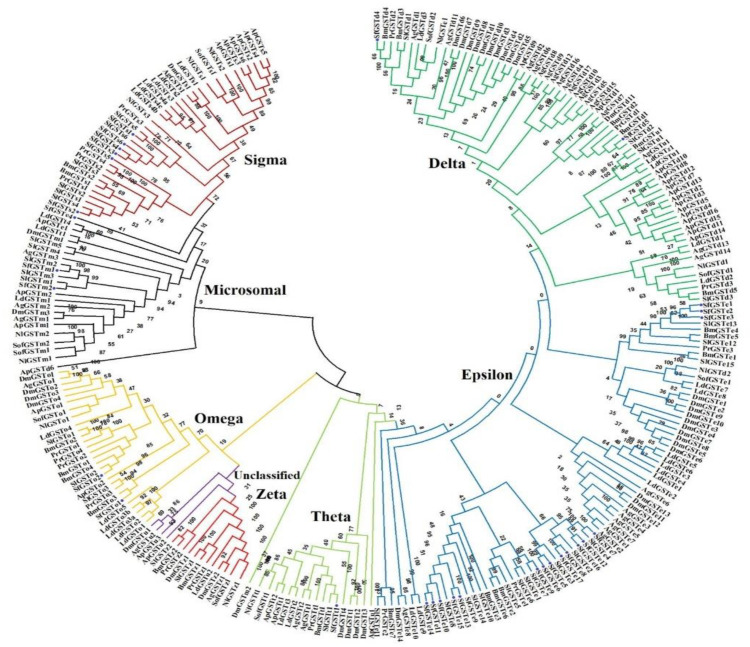
Phylogenetic analysis of SfGSTs in *S. frugiperda*. The phylogenetic tree was constructed using a neighbor-joining method to analyze the amino acid sequences of insect GSTs. Sl, *Spodoptera litura*; Pr, *Pieris rapae*; Bm, *Bombyx mori*; Nl, *Nilaparvata lugens*; Sf, *Sogatella furcifera*; Dm, *Drosophila melanogaster*; Ld, *Leptinotarsa decemlineata*; Ap, *Acyrthosiphon pisum*.

A total of 31 SfGST genes were discovered in the *S. frugiperda* genome and transcriptome, involving 27 cytosolic and 3 microsomal SfGSTs, designated SfGSTd1–SfGSTt1 (Table 2). Based on phylogenetic analyses and protein sequence comparisons with other insect GSTs, such as *Spodoptera litura*, *Pieris rapae*, *Nilaparvata lugens*, *Sogatella furcifera*, *Leptinotarsa decemlineata*, *Acyrthosiphon pisum*, *Anopheles gambiae*, and *Bombyx mori*, 28 cytosolic CsGSTs were further classified into 5 classes depending on NCBI blast and phylogenetic analysis, including 2 deltas (SfGSTd1 and SfGSTd2); 17 epsilons (SfGSTe1 to SfGSTe17); 2 omegas (SfGSTo1 and SfGSTo2); 5 sigmas (SfGSTs1 to SfGSTs6); 1 theta (SfGSTt1); and 3 microsomal genes (SfGSTm1, SfGSTm2, and SfGSTm3) (Table 3). A phylogenetic examination of GSTs from other insect orders revealed that SfGSTs have a high degree of similarity with the GSTs of *S. litura* and *B. mori*, and the homologous genes of various insects are grouped together in the same clade (Figure 1). According to a sequencing study of the SfGSTs genes, the 31 SfGSTs had full open reading frames (ORF) that encoded 63-282 amino acids with protein molecular masses ranging from 7.05 to 94.38 kDa.

### 3.3. Expression Profiling of sfGSTs in S. frugiperda Larvae Exposed to Tested Insecticides

**Figure 2:**
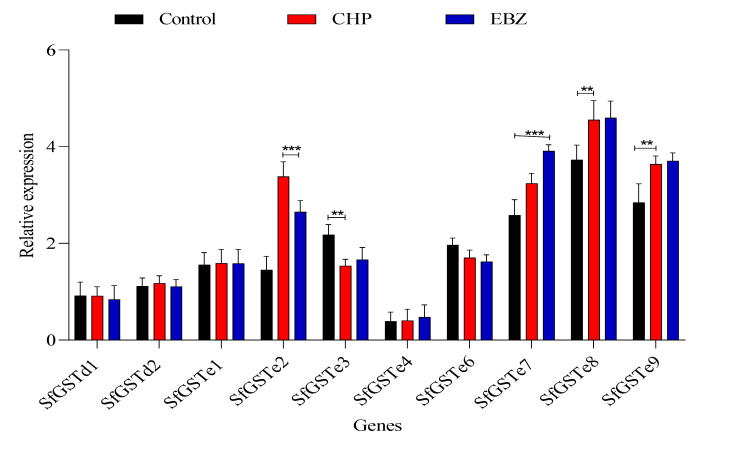

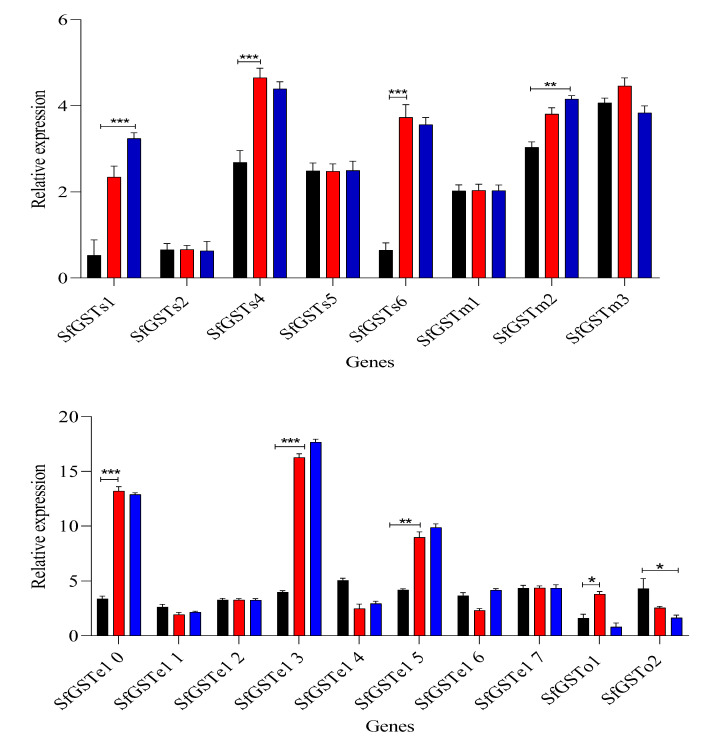
Relative expression levels of SfGSTs in larvae exposed to an LC_50_ of emamectin benzoate (EBZ) and chlorantraniliprole (CHP). Dunnett’s tests were performed to compare the gene expression of the tested insecticides with the corresponding control group (* *p* < 0.05, ** *p* < 0.01, *** *p* < 0.001).

SfGST transcript levels were measured in larvae exposed to LC_50_ concentrations of these insecticides to evaluate whether SfGST expression responds to CHP and EBZ (Figure 2). The expression of the 28 SfGSTs showed that 11 SfGSTs (SfGSTe2, SfGSTe7, SfGSTe8, SfGSTe9, SfGSTe10, SfGSTe13, SfGSTe15, SfGSTs1, SfGSTs4, SfGSTs6, and SfGSTm2) were significantly upregulated under LC_50_ of CHP and EBZ compared with the control. Conversely, five genes (SfGSTe3, SfGSTe6, SfGSTe11, SfGSTe14, and SfGSTo2) were significantly downregulated after exposure to LC_50_ of CHP and EBZ. The mRNA levels of sfGSTo1 and sfGSTm3 decreased with EBZ by 0.81 and 3.83 fold but increased with CHP by 3.81 and 4.46 fold compared with the control. Meanwhile, the mRNA level of sfGSTe16 increased by 4.18 fold with EBZ and decreased by 2.32 fold with CHP. Remarkably, sfGSTe10 and sfGSTe13 showed the highest relative expression with 13.21- and 16.27-fold increases for CHP and 12.89- and 17.66-fold increases for EBZ, respectively.

### 3.4. Molecular Docking Analysis

Based on relative expression experiments, a molecular docking analysis using SfGSTe10 and SfGSTe13 as the most upregulated genes and SfGSTs1 and SfGSTe2 as the least upregulated genes of *S. frugiperda* larvae was built using homology modeling. The docking analysis showed that EBZ had a higher binding affinity for SfGSTe13 (−26.85 kcal/mol), followed by SfGSTe10 (−24.41 kcal/mol), SfGSTs1 (−23.16 kcal/mol), and SfGSTe2 (−19.01 kcal/mol), whilst CHP had a higher binding affinity with SfGSTe13 (−26.78 kcal/mol), followed by SfGSTe10 (−26.72 kcal/mol), SfGSTe2 (−19.52 kcal/mol), and SfGSTs1 (−18.45 kcal/mol) (Table 4).

**Figure 3:**
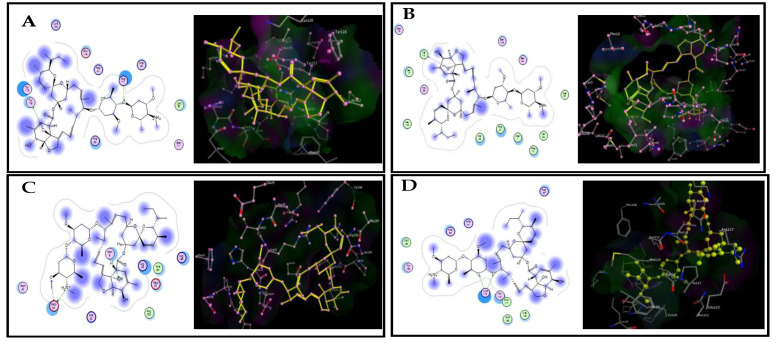
Docking view of the binding interactions of emamectin benzoate (EBZ) within four receptors, (**A**) *SfGSTe10*, (**B**) *SfGSTe13*, (**C**) *SfGSTs1*, (**D**) *SfGSTe2*), in *S. frugiperda*. **Left**: two-dimensional interaction diagram of insecticide–receptor complexes. **Right**: the 3D complex structure and ligand bonds are depicted by yellow lines.

As a ligand, EBZ penetrates deep into the hydrophobic pocket in *SfGSTe10* (4.47 Å) through two bonds (H-bonds), with Arg 111 and an H-pi bond with Tyr 116, surrounded by residual Lys 120, Tyr 115, Glu 115, Arg 41, His 40, Leu 25, Ser 11, and His 52. EBZ links to the active site of *SfGSTe13* via an H-pi bond with Val 15, surrounded by more residues of Ser 140, Ser 49, Met 99, Val 90, Phe 10, Met 1, Pro 97, Val 90, Lau 100, Thr 99, and Lau 17. EBZ connects to the active sites of *SfGSTs1* (1.49 Å) via H– bonds with Asn 9 and two bonds with Asp 34, as well as van der Waals interactions with a large number of amino acids: Asn 45, His22, Arg 22, Ala 197, Arg 198, and Asp 22. In *SfGSTe2*, EBZ is linked by two bonds with Lys 112 and via van der Waals interactions with the following amino acids: Leu 36, Tyr 120, His 53, His 41, Arg 127, Thr 113, Pro 112, Val 116, and Ala 158 (Figure 3).

**Figure 4:**
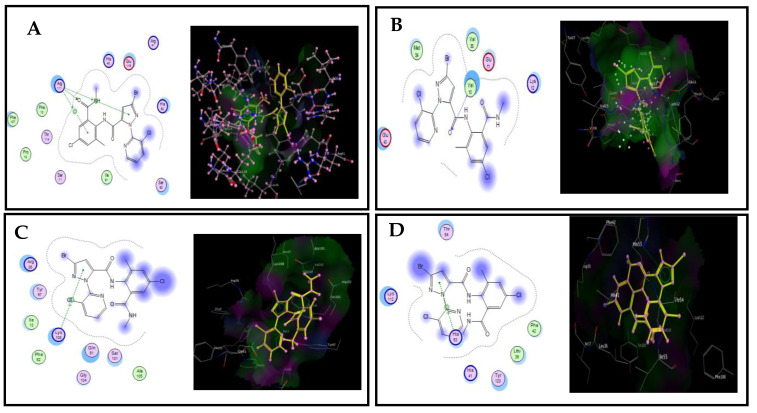
Docking view of the binding interactions of chlorantraniliprole (CHP) within four receptors, (**A**) *SfGSTe10*, (**B**) *SfGSTe13*, (**C**) *SfGSTs1*, (**D**) *SfGSTe2*), in *S. frugiperda*. **Left**: two-dimensional interaction diagram of insecticide–receptor complexes. **Right**: the 3D complex structure and ligand bonds are depicted by yellow lines.

An amino acid (Arg 111) binds to CHP through three bonds (two H-pi bonds and one H- bond as a hydrophobic interaction) in the *SfGSTe10* receptor with a distance of 2.94 Å and is in a pocket formed by residual Phe 107, Phe 10, Pro 12, Thr 114, Ser 11, Lle 61, Ser 53, His 52, Arg 41, Glu 119, and His 40. CHP connects to the active sites of *SfGSTe13* via H– bonds with Val 13 (2.36 Å) and van der Waals interactions with many amino acids, including Glu 40, Met 39, Val 38, and Glu 11. Meanwhile, CHP connects to the active sites of *SfGSTs1* via an H-pi bond with Lys 108 (4.61 Å) and eight amino acids (Arg 35, Tyr 97, Lle 13, Phe 52, Gin 51, Gly 104, Ser 101, and Ala 105) in the active site through van der Waals interactions. Likewise, in *SfGSTe2*, this insecticide connects via an H-pi bond with His 53 (4.42 Å) and is surrounded by residual Thr 54, Lys 112, His 41, Tyr 120, Leu 35, and Phe 42 (Figure 4).

## 4. Discussion

*Spodoptera frugiperda* is an insect that causes huge agricultural losses all over the world [50]. It has remarkable long-distance flight ability, a high dispersal ability, and a high potential for intensive migratory behavior. Consequently, it is necessary to intervene with chemical controls to limit the spread of this insect. In this study, EBZ and CHP were proved to be toxic to *S. frugiperda* larvae with LC_50_ values of 0.029 and 1.250 mg/L at 24 h. EBZ is a significant Avermectin family macrocyclic lactone pesticide with outstanding effectiveness against lepidopteran pests [51]. EBZ causes DNA damage and apoptosis in *S. frugiperda*’s sf-9 cell line [52]. Furthermore, EBZ produces a hyperpolarized cell by attaching to glutamate-gated chloride channels and causing an inflow of chloride ions [53]. CHP is highly effective against lepidopteran insects and was the first ryanodine receptor insecticide to be developed from a novel chemical class [54]. This outcome was in line with Zhao, et al. [55], who showed that the LC_50_ values of CHP against *S. frugiperda* ranged from 0.849 mg/L in Xuzhou to 3.446 mg/L in Dongtai. The LC_50_ values of EBZ against *S. frugiperda* ranged from 0.019 mg/L in Yichang to 0.041 mg/L in Dehong. Moreover, the LC_50_ value of CHP was 0.068 µg/mL against third-instar *S. frugiperda* larvae [56]. Interestingly, EBZ achieved toxicity with an LC_50_ value of 0.383 against the third-instar larvae of *S. frugiperda* [57]. Furthermore, the LD_50_ values of CHP and EBZ were 0.410 and 0.355 µg/g against *S. frugiperda* larvae [58].

GSTs are involved in detoxifying endogenous and external chemical compounds and are linked to the development of pesticide resistance [59]. Based on genomic data, GSTs have been identified from a range of insects; however, the number and classification of GST genes vary between insect species. For example, 17 GSTs genes were discovered in Pieris rapae [45], 22 GSTs were discovered in *Plutella xylostella* [14], 32 GSTs were discovered in *Acyrthosiphon pisum* [47], 35 GSTs were discovered in *Anopheles gambiae* [48], and 25 GSTs were discovered in *Cnaphalocrocis medinalis* [60]. In the present study, 31 sfGST genes were identified in the *S. frugiperda* genome and transcriptome, which had similar number and classification distributions to those of 37 lepidopteran GSTs in *Spodoptera litura* [44] and 24 GSTs in *Bombyx mori* [49]. Therefore, GST levels differ widely between insect species.

We used phylogenic tree analysis to determine GST classifications. As a result, 31 sfGSTs were discovered and classified as delta, epsilon, omega, sigma, theta, and microsomal. The two largest categories identified in this insect are sigma and epsilon. GSTs from these two classes frequently play detoxifying roles and have been linked to insecticide resistance [18]. For example, *SfGSTe7* was discovered in the same lineage as *SlGSTe2*, an enzyme implicated in carbaryl, DDT, and deltamethrin detoxification [44]. Likewise, In the epsilon clade, *SfGSTe8* was found to be closely related to *SlGSTe3*, and it was marginally upregulated in *S. litura* by carbaryl and DDT [61]. Moreover, *SfGSTs1* and *SfGSTs4* appeared in the same clade as BmGSTs2, and the expression of the BmGSTs2 gene increased in the midgut after exposure to the herbicide glyphosate and the insecticide permethrin, reaching a peak at 6 to 12 h in *B. mori* [62].

Our finding suggests that 11 out of 28 genes showed the highest relative expression in *S. frugiperda* against CHP and EBZ. This could be because GSTs share a set of detoxifying enzyme systems for different kinds of pesticides [2,59]. This result is in agreement with Hu, et al. [63], who showed that GSTe6 was upregulated against CHP in *Spodoptera exigua*. Moreover, abamectin and CHP significantly upregulated PrGSTs1 in *P. rapae* [45]. Furthermore, 9 GSTs out of 16 identified were upregulated after different periods and doses of malathion exposure, while 3 GSTs were upregulated by β-cypermethrin exposure in *Bactrocera dorsalis* [64]. In addition, the mRNA levels of GSTe1, 3, 10, and 15 increased in *S. litura* under chlorpyrifos compared with the control [44]. Additionally, EBZ significantly upregulated GmGSTs1 in *Grapholita molesta* [65].

In contrast, our data showed that the transcript levels of five genes (*SfGSTe3*, *SfGSTe6*, *SfGSTe11*, *SfGSTe14*, and *SfGSTo2*) were significantly downregulated under CHP and EBZ stress. An adaptive mechanism in insects that lowers the overall activity of GST enzymes to avoid excessive GST activity from a depleted supply of GSH may involve downregulating a subset of GSTs [1]. The expression levels of PxGSTd1, PxGSTd2, and PxGSTd4 were significantly decreased in *P. xylostella* under acephate stress [66]. Moreover, SfGSTo1 and SfGSTt1 were slightly downregulated in *Sogatella furcifera* under chlorpyrifos stress [46].

Molecular docking was applied to predict the binding sites for proteins. Structural modeling aids in understanding the binding mechanisms between any protein and any ligand [67]. Protein–ligand docking was carried out using the MOE and an induced fit technique with a fixed receptor and a flexible ligand [68]. A protein–ligand complex’s binding interaction can be identified using the docking process, as well as binding geometry and other interactions [69]. Small compounds have frequently been docked to target enzymes using this technique [70]. Our findings showed that four receptors (*SfGSTe10*, *SfGSTe13*, *SfGSTs1*, and *SfGSTe2*) were targets of CHP and EBZ. Our data showed the binding affinity between CHP and EBZ to be high with *SfGSTe10* and *SfGSTe13* and low with *SfGSTs1* and *SfGSTe2*, indicating the potential of *SfGSTe10* and *SfGSTe13* to metabolize CHP and EBZ more than *SfGSTs1* and *SfGSTe2*. This is evident in the compatibility of the gene expression data with molecular docking analysis (the lowest binding energy with ligands), which showed that the binding affinity between CHP and EBZ is high with *SfGSTe10* and *SfGSTe13*, and low with *SfGSTs1* and *SfGSTe2*. This result may be because the amino acids in the receptor are linked by double or triple bonds, and the docking binding affinity is high with lower binding energy [71,72]. Additionally, the type and position of amino acids in the active site of GSTs (the G-site and H-site) play important roles in insecticide binding affinity and catalytic functions [73]. The enzyme catalyzes the nucleophilic attack by the glutathione and its conjugation with the substrate, making the substrate less reactive and more soluble, and, therefore, it is easier for it to excrete GSTs, detoxifying insecticides by catalyzing nucleophilic attacks from the thiol group in reduced glutathione (GSH) in a wide range of electrophilic substrates [8,19]. In addition, GSTs may participate in the passive non-catalytic binding of substrates and sequestration, which prevents insecticides from binding to their target proteins [20]. Consequently, evidence was provided that *SfGSTe10*, *SfGSTe13*, *SfGSTs1*, and *SfGSTe2* catalyzed to conjugate with CHP and EBZ and identified the possible site of binding amino acids with insecticides. A study showed that the activity of recombinant TcGSTm02 in *Tetranychus cinnabarinus* could be inhibited by cyflumetofen, and the enzyme catalyzed the conjugation of GSH into cyflumetofen [74]. In another study, the mechanism of detoxification by GSTD2 in *D. melanogaster* was revealed by its strong affinity toward isothiocyanate and catalyzing the conjugation between GSH and isothiocyanate [75]. Likewise, *Bombyx mori*, the antenna-specific BmGSTD4, had high GSH-conjugating activity toward 1-chloro-2 and 4-dinitrobenzene (CDNB), indicating its potential role in the metabolism of xenobiotics [76]. Moreover, higher binding affinities between pesticides and enzymes were observed with lower binding energies [77,78]. Finally, the molecular docking is considered complementary to the aforementioned results at the applied level, not just the research level, because this part shows the effect of insecticides and their contents on insect proteins and enzymes, which are necessary to know the extent of the insect’s ability to show the resistance to these pesticides in the long term [79]. On the environmental level, knowing which of the chemical bonds within the insecticides is more closely related to the amino acids within the target protein of the target insect helps us design more targeted pesticides for specific organisms without any side effects on other organisms in the surroundings, or in other words, designing more specialized chemical pesticides at the genetic level for target organisms.

## 5. Conclusions

In this study, bioassay results showed that EBZ and CHP have a toxic effect on the third-instar larvae of *S. frugiperda*. In addition, 31 SfGST genes were identified from *S. frugiperda* by analyzing previously published transcriptome data, and the phylogenetic relationships of SfGSTs were investigated. SfGST genes showed different expression profiles following exposure to CHP and EBZ. The obtained data showed that SfGST genes are related to EBZ and CHP detoxification in *S. frugiperda*. Molecular docking revealed that EBZ and CHP have a high binding affinity with SfGSTe13 compared with other genes. Our findings focused on the roles of GSTs in EBZ and CHP detoxification in *S. frugiperda*.

## Figures and Tables

**Table 1:** Bioassay of EBZ and CHP against the third-instar of *Spodoptera frugiperda* after 24 h of treatment.

Compounds	Toxicity Regression Equation	LC_50_ (mg/L)	95% Fiducial Limits (mg/L)	r	df
EBZ	y = 0.059x + 3.2355	0.029	0.019–0.045	0.97	4
CHP	y = 0.058x + 2.6388	1.250	0.973–1.455	0.96	4

**Table 2:** Details of the 31 GSTs identified in *Spodoptera frugiperda*.

Group	Gene Name	ORF (bp)	Protein (aa)	Molecular Weight (kDa)	Theoretical pI	NCBI ID
Delta	*SfGSTd1*	723	240	27.23	5.64	MZ673611
	*SfGSTd2*	651	216	24.22	6.90	MZ673619
Epsilon	*SfGSTe1*	594	197	23.01	5.75	MZ673615
	*SfGSTe2*	654	217	25.17	6.23	MZ673616
	*SfGSTe3*	654	217	25.14	6.53	MZ673617
	*SfGSTe4*	348	115	94.38	5.71	MZ673620
	*SfGSTe5*	291	96	11.16	6.26	MZ673621
	*SfGSTe6*	669	222	25.11	7.81	MZ673622
	*SfGSTe7*	693	230	25.75	8.42	MZ673623
	*SfGSTe8*	429	142	15.95	6.90	MZ673625
	*SfGSTe9*	432	143	23.83	6.72	MZ673626
	*SfGSTe10*	657	218	24.98	6.97	MZ673629
	*SfGSTe11*	657	218	25.06	7.10	MZ673630
	*SfGSTe12*	420	139	15.73	6.90	MZ673632
	*SfGSTe13*	474	157	18.05	5.19	MZ673633
	*SfGSTe14*	540	179	20.59	5.86	MZ673635
	*SfGSTe15*	657	218	24.90	6.96	MZ673636
	*SfGSTe16*	420	139	15.76	6.90	MZ673637
	*SfGSTe17*	192	63	7.05	5.44	MZ673638
Omega	*SfGSTo1*	723	240	28.77	7.01	MZ673610
	*SfGSTo2*	849	282	32.48	7.01	MZ673624
Sigma	*SfGSTs1*	618	205	23.98	6.98	MZ673614
	*SfGSTs2*	615	204	23.09	4.92	MZ673618
	*SfGSTs3*	639	212	24.19	6.11	MZ673628
	*SfGSTs4*	639	212	20.07	5.44	MZ673631
	*SfGSTs5*	108	35	10.09	4.82	MZ673634
	*SfGSTs6*	633	210	23.87	5.61	MZ673639
Microsomal	*SfGSTm1*	516	171	16.59	9.62	MZ673612
	*SfGSTm2*	456	151	16.64	9.79	MN480699
	*SfGSTm3*	453	150	16.64	9.65	MZ673627
Theta	*SfGSTt1*	687	228	26.42	7.66	MN480695

ORF: Open reading frame.

**Table 3:** Comparison of GST genes in various insect species. Data are collated from [10,15,44,45,46,47,48,49].

Species	Delta	Epsilon	Omega	Sigma	Theta	Zeta	Unclassified	Microsomal	Total
*Spodoptera frugiperda*	2	17	2	6	1	0	0	3	31
*Spodoptera litura*	3	15	3	6	1	2	1	5	36
*Pieris rapae*	3	3	4	4	1	2	0	0	17
*Nilaparvata lugens*	2	1	1	3	1	0	1	2	11
*Sogatella furcifera*	2	1	1	1	1	1	0	2	9
*Leptinotarsa decemlineata*	3	10	5	4	4	1	2	1	30
*Drosophila melanogaster*	11	14	4	1	4	2	1	3	40
*Acyrthosiphon pisum*	16	1	2	6	2	0	2	3	32
*Anopheles gambiae*	17	8	1	1	2	1	2	3	35
*Bombyx mori*	5	7	4	2	1	2	0	1	22

**Table 4:** Docking results for chlorantraniliprole (CHP) and emamectin benzoate (EBZ) within four modeled active sites of *S. frugiperda*.

Genes	EBZ		CHP	
	Binding Energy (kcal/M)	RMSD (A ◦)	Binding Energy (kcal/M)	RMSD (A ◦)
*SfGSTe10*	−24.41	4.47	−26.72	2.94
*SfGSTe13*	−26.85	2.96	−26.78	2.36
*SfGSTs1*	−23.16	1.49	−18.45	4.61
*SfGSTe2*	−21.19	2.36	−19.52	4.42

RMSD: root-mean-square deviation.

## Data Availability

All data and materials are included in the manuscript.

## Supplementary Materials

The following supporting information can be downloaded at https://www.mdpi.com/article/10.3390/toxics11060542/s1: Table S1: primer sequences of qRT-PCR.
