# Supplementary material for: Identification and Characterization of Glutathione S-transferase Genes in Spodoptera frugiperda (Lepidoptera: Noctuidae) under Insecticides Stress
Source: Toxics. 2023 Jun 19;11(6):542. doi: 10.3390/toxics11060542 (PMC10303185; doi:10.3390/toxics11060542)
Supplement: Supplementary file 1 [file toxics-11-00542-s001.zip › toxics-2439116-supplementary.pdf]

Table S1. Primer sequences of qRT-PCR.

Gene ID	Primer		Length (bp)	Tm.	Product size
SfGSTd1	F	CAGTTAGACGAGGCCCTTGG	20	60	83
	R	TCGGCGATGGTGAGATTGTC	20	60	
SfGSTd2	F	AGGTGCAATAAGTGCTCAGTA	21	60	107
	R	GGAAGCCAAATAGAGTGCAAACA	23	60	
SfGSTe1	F	AACGGGCCATTGTAGACCAG	20	60	177
	R	CAAGCCAGGGTCCAGAGAAG	20	60	
SfGSTe2	F	ATGGTGAGACGGCATTCAGG	20	60	88
	R	AGCCAGGGTCCAGAGAAGAA	20	60	
SfGSTe3	F	GATTCAACGCGAACACTTCA	20	60	230
	R	TTTTCAATGCTGCGAAGAGA	20	60	
SfGSTe4	F	GCAATCTCTGGTCACAGCAA	20	60	242
	R	GGCTCCGGGGATATATCAAT	20	60	
SfGSTe6	F	CCCGGACTACCTGAAGTTGA	20	60	199
	R	GAGTATCCCGGTGTCGAAGA	20	60	
SfGSTe7	F	ATGCTTGGCCTACAAATGGA	20	60	150
	R	CTTCATTATGGCGTGGCTTT	20	60	
SfGSTe8	F	CGTCGGCATATTCTTCATCA	20	60	231
	R	AGATTTTCTGGGGACCTCGT	20	60	
SfGSTe9	F	ACAGGACAGCCATGCAATCA	20	60	289
	R	CGATATGTCGGCCAAGGTCA	20	60	
SfGSTe10	F	AGAAGCCATTGGTTTTGTGG	20	60	162
	R	TCTAACCAGGCAACCGTCTT	20	60	
SfGSTe11	F	GACACCTACGGAAGGGATGA	20	60	234
	R	ATCCAAGGCGATGAACTTTG	20	60	

SfGSTe12	F	AGTCGGGGTGAGTGAGACAC	20	60	196
	R	ATTGCCGTACATTGGATGGT	20	60	
SfGSTe13	F	GCTGACACCTACGGAAAGGA	20	60	249
	R	AATGGTGACAAGGTCCAAGG	20	60	
SfGSTe14	F	AAGCCATTGGTTTTGTGGAG	20	60	175
	R	TGAAACACTGGGACCAACAA	20	60	
SfGSTe15	F	AGAAGCCATTGGTTTTGTGG	20	60	162
	R	TCTAACCAGGCAACCGTCTT	20	60	
SfGSTe16	F	AGTCGGGGTGAGTGAGACAC	20	60	196
	R	ATTGCCGTACATTGGATGGT	20	60	
SfGSTe17	F	CACAAGATGGCGCCGATAC	19	60	102
	R	GTCCCTGACTTCGAGTTCCA	20	60	
SfGSTo1	F	TGGACATGAACCCCTATGGT	20	60	193
	R	CAAATCACCACGCTCTCAAA	20	60	
SfGSTo2	F	AGCGACCTCTCCTCCCTAAG	20	60	242
	R	CCAGGGCCAGATCATGTAGT	20	60	
SfGSTs1	F	GCCGCTGTGTACACCATCTA	20	60	195
	R	GACGAACTCCACCCAGGATA	20	60	
SfGSTs2	F	ATCGACCAGAACGTTGACCT	20	60	170
	R	ACATGGCCGTTGTTCTTGAC	20	60	
SfGSTs4	F	CATAGAGAGCATCGTATTCACC	22	60	202
	R	TACAAAGTCACCCCAGGAG	19	60	
SfGSTs5	F	TGGTTACGTCGCTTTAGGAAA	21	60	112
	R	GGCTGGGTACTTCTCTCCAA	20	60	
SfGSTs6	F	CTGAAGGGCAAGACTGCGTA	20	60	201

	R	ACAATGTGTGCGCTTTGGTC	20	60	
SfGSTm1	F	CACAGTGAACAAGCGACTCG	20	60	203
	R	AAGCCATGCCCAGAACTTTG	20	60	
SfGSTm2	F	TCAACCGAGCACAAACAGAG	20	60	161
	R	GGCTCGGGTAGTACCTGTCA	20	60	
SfGSTm3	F	TCCAGTGCGCTGTGATTTCT	20	60	175
	R	CAGTTGCCTACCACGACAGT	20	60	
EF1α	F	TGGTGACTCCAAGAACAACC	20	60	175
	R	CGGTGGATTTACCAGTACGA	20	60	
RPS18	F	GACTGGTTCCTCAACAGGCA	20	60	143
	R	CCCCAGTAGTGACGCATACC	20	60	
